# Developing and validating a drug recommendation system based on tumor microenvironment and drug fingerprint

**DOI:** 10.3389/frai.2024.1444127

**Published:** 2025-01-08

**Authors:** Yan Wang, Xiaoye Jin, Rui Qiu, Bo Ma, Sheng Zhang, Xuyang Song, Jinxi He

**Affiliations:** ^1^Department of Medical Oncology, General Hospital of Ningxia Medical University, Yinchuan, China; ^2^General Thoracic Surgery, General Hospital of Ningxia Medical University, Yinchuan, China

**Keywords:** tumor microenvironment, drug fingerprint, IC_50_, MFI, Best Overall Response

## Abstract

**Introduction:**

Tumor heterogeneity significantly complicates the selection of effective cancer treatments, as patient responses to drugs can vary widely. Personalized cancer therapy has emerged as a promising strategy to enhance treatment effectiveness and precision. This study aimed to develop a personalized drug recommendation model leveraging genomic profiles to optimize therapeutic outcomes.

**Methods:**

A content-based filtering algorithm was implemented to predict drug sensitivity. Patient features were characterized by the tumor microenvironment (TME), and drug features were represented by drug fingerprints. The model was trained and validated using the Genomics of Drug Sensitivity in Cancer (GDSC) database, followed by independent validation with the Cancer Cell Line Encyclopedia (CCLE) dataset. Clinical application was assessed using The Cancer Genome Atlas (TCGA) dataset, with Best Overall Response (BOR) serving as the clinical efficacy measure. Two multilayer perceptron (MLP) models were built to predict IC_50_ values for 542 tumor cell lines across 18 drugs.

**Results:**

The model exhibited high predictive accuracy, with correlation coefficients (*R*) of 0.914 in the training set and 0.902 in the test set. Predictions for cytotoxic drugs, including Docetaxel (*R* = 0.72) and Cisplatin (*R* = 0.71), were particularly robust, whereas predictions for targeted therapies were less accurate (*R* < 0.3). Validation with CCLE (MFI as the endpoint) showed strong correlations (*R* = 0.67). Application to TCGA data successfully predicted clinical outcomes, including a significant association with 6-month progression-free survival (PFS, *P* = 0.007, AUC = 0.793).

**Discussion:**

The model demonstrates strong performance across preclinical datasets, showing its potential for real-world application in personalized cancer therapy. By bridging preclinical IC_50_ and clinical BOR endpoints, this approach provides a promising tool for optimizing patient-specific treatments.

## 1 Introduction

In recent years, significant advancements have been made in novel cancer treatment modalities, notably in the realms of immunotherapy and targeted therapy (Zhu et al., 2018; Xue et al., 2020). While immunotherapy has transformed the landscape of cancer treatment, instances of successful outcomes remain relatively sparse (Fan et al., 2021). Immunotherapy typically necessitates the identification of specific biomarkers within a patient's tumor, such as PD-1 and PD-L1, to predict its efficacy (Dong et al., 2023; Shergold et al., 2019). However, the positivity rates for these biomarkers typically fall below 10%. Furthermore, though targeted therapies for cancer have offered hope in the field of oncology, their effectiveness is plagued by multiple limitations (Zhou et al., 2021). Tumor heterogeneity presents a substantial challenge to treatment, as these cancer cells may mutate or selectively adapt under therapeutic pressure, altering the expression or function of the target and rendering targeted therapy ineffective (Viallard and Larrivée, 2017; Deepak et al., 2020). This implies that in the coming decade, the majority of patients may not benefit from such treatments. Therefore, chemotherapy remains the primary approach to cancer treatment at present.

Due to the broad-spectrum cytotoxicity of chemotherapy drugs, they exhibit antitumor effects across a variety of cancer types (El-Hussein et al., 2021; Salas-Benito et al., 2021). This characteristic endows chemotherapy with wide applicability in the treatment of tumors of various types and stages. However, the diversity of cancer and varying patient responses to different therapies present a significant challenge in cancer treatment. While chemotherapy can effectively target cancer cells, there are noticeable individual differences in treatment outcomes and tolerances. Personalized medicine has become a paramount objective in the field of oncology to enhance treatment efficacy and reduce unnecessary side effects (Masood and Wu, 2023; Pasetto and Lu, 2023). Hence, there is an urgent need for a method to more accurately identify which patients will benefit from these drug therapies, thereby avoiding unnecessary treatments and alleviating patient discomfort.

In the early stages of single-molecule analysis, researchers attempted to predict patient responses to chemotherapy drugs by analyzing specific biomarkers such as ERCC1. However, years of effort have shown that patient responses to chemotherapy drugs are an extremely complex process influenced by multiple variables (Irajizad et al., 2022). Importantly, this process requires substantial data support. In the absence of large-scale data models, data screening, organization, and analysis are time-consuming tasks, and manual screening is prone to errors.

With the advancement of large-scale data and computational capabilities, researchers are now able to leverage multi-level information from patients, including genomics and transcriptomics (Mann et al., 2021; Chen et al., 2022; Li R. et al., 2022). Building upon this foundation, researchers have developed models applicable to various scenarios for a more comprehensive and precise evaluation of individual sensitivity to chemotherapy drugs (Huang et al., 2023). Among various computational models, deep learning methods have received significant attention and recognition for their outstanding ability to extract complex patterns and relationships from diverse datasets (Li Y. et al., 2022). Deep learning methods utilize neural network architectures with multiple interconnected nodes, mimicking the complex structure and functions of the human brain (Ho et al., 2020). This architecture enables models to efficiently process and analyze various data representations, including genomic data, clinical data, and imaging data, thereby aiding in the comprehensive evaluation of multiple factors influencing drug sensitivity in cancer patients (Vatansever et al., 2021).

This study aims to fully harness the potential of DL to establish a model that predicts the utility of chemotherapy drugs in cancer treatment. Initially, we meticulously selected 407 cell lines from the Genomics of Drug Sensitivity in Cancer (GDSC) database to construct and validate predictive models for drug sensitivity. Independent validation was conducted using 93 cell lines from the Cancer Cell Line Encyclopedia (CCLE) database. Furthermore, we applied the model to test its performance using 105 human tissue samples from The Cancer Genome Atlas (TCGA). The results demonstrate that this model accurately predicts drug sensitivity. It not only provides physicians with a more reliable decision support tool for precise, personalized treatment planning but also alleviates the physical and economic burdens imposed on patients by treatment. The success of this research holds the potential to overcome previous challenges, offering more effective treatment choices for cancer patients and further advancing the field of precision medicine.

## 2 Materials and methods

### 2.1 Database and data cleaning

We employed the GDSC database (https://www.cancerrxgene.org/) for model development and validation. The CCLE database (https://sites.broadinstitute.org/ccle) served as an independent validation dataset, while TCGA human tissue samples were used to assess the real-world application of the model.

Given our ultimate goal of applying the model within the TCGA dataset, data cleaning became crucial for our research. Consequently, we utilized pertinent data from the TCGA database to infer the necessary cell line data. We initiated this process by meticulously screening patient treatment data, with a particular focus on selecting patients who underwent single-agent therapy, using their BOR as the endpoint. Therefore, we selected 542 GDSC cell lines from GDSC, 93 cases from CCLE, and 105 patient data from TCGA who received single-agent drug treatment, encompassing 12 tumor types and 18 drug categories.

The TCGA-PANCANCER dataset was obtained from UCSC Xena (https://xenabrowser.net/datapages/) and includes phenotype data, somatic mutation status (SNV), and gene expression profiles (GEP). In total, 8,347 patients had complete SNV and GEP data available. BOR data for TCGA patients were retrieved from a previous publication (Ding et al., 2016), resulting in a subset of 1,197 patients. After intersecting patient IDs, 404 patients who received monotherapy had complete SNV, GEP, and BOR data. This preprocessed TCGA cohort spans 54 drugs and 24 cancer types, which were used for further data filtering. In the GDSC dataset, 978 cell lines were collected initially. By intersecting this data with the preprocessed TCGA cohort, we filtered down to 18 drugs and 12 cancer types. After considering SNV and GEP, a final set of 542 GDSC cell lines were selected. Based on the final drug and cancer-type selection, the TCGA cohort was filtered to 105 patients, and cell lines in the CCLE dataset were reduced from 1,826 to 93 cases (Supplementary Figure S1).

Data normalization primarily followed the procedures established by the respective databases. We used three types of explanatory variables: phenotypic features (sex, age, cancer type), SNV (single nucleotide variants), and GEP. Phenotypic features were converted into dummy variables, i.e., binary variables with values of 0 or 1. For SNV features, genes with non-silent mutations were labeled as 1, and 0 otherwise. For GEP features, we used the normalized expression format, transcripts per million (TPM). For response variables (y), we utilized three types: IC_50_ from GDSC, MFI from CCLE, and BOR from TCGA. IC_50_ values were log-transformed using the natural logarithm. MFI values were used directly in their normalized form, specifically the replicate-collapsed log-fold-change values (Corsello et al., 2020). For BOR, we categorized the response into binary outcomes: PD (progressive disease) and non-PD.

All three datasets (GDSC, CCLE, and TCGA) employed consistent data transformation methods. The response variables (y) were processed into a one-dimensional format, representing the drug response for each sample-drug pair. For instance, in the GDSC dataset, the y value was the log-transformed IC_50_ for each cell line-drug pair, resulting in a vector with 9,756 values (corresponding to 542^*^18). The explanatory variables (X) were transformed accordingly. For example, the SNV data in GDSC was reshaped into a matrix with 9,756 rows and 776 columns, where each row represents a cell line-drug pair, and each column represents the mutation status of the selected genes.

Drug molecular structures were represented using SMILES (Simplified Molecular Input Line Entry System), which were sourced from PubChem (https://pubchem.ncbi.nlm.nih.gov/). The SMILES were converted into Morgan fingerprints (also known as Extended Connectivity Fingerprints, ECFP) using RDKit software (version 2022.09.1), a widely used cheminformatics tool for circular fingerprint generation.

### 2.2 Model construction

Our methodology, mainly based on small molecule drugs, focuses on cytotoxicity, using IC_50_ as a key indicator of cytotoxicity in the GDSC database. The response variables in CCLE are slightly more complicated (Corsello et al., 2020). In brief, a barcode is introduced for each cell line. After drug treatment, the fluorescence intensity of each barcode, called luminex MFI, is measured. This fluorescence intensity is then compared with the MFI of negative controls treated with dimethylsulfoxide (DMSO). The resulting log fold change of MFI is used as the final drug sensitivity indicator (referred to as MFI hereafter in our text). The smaller the MFI, the more sensitive to the drug. We used known genomic mutations of tumor-driving genes, transcriptomic expression data, and essential clinical characteristics of cell lines, including tumor type, patient age, and gender, as explanatory variables (X).

**Figure 1:**
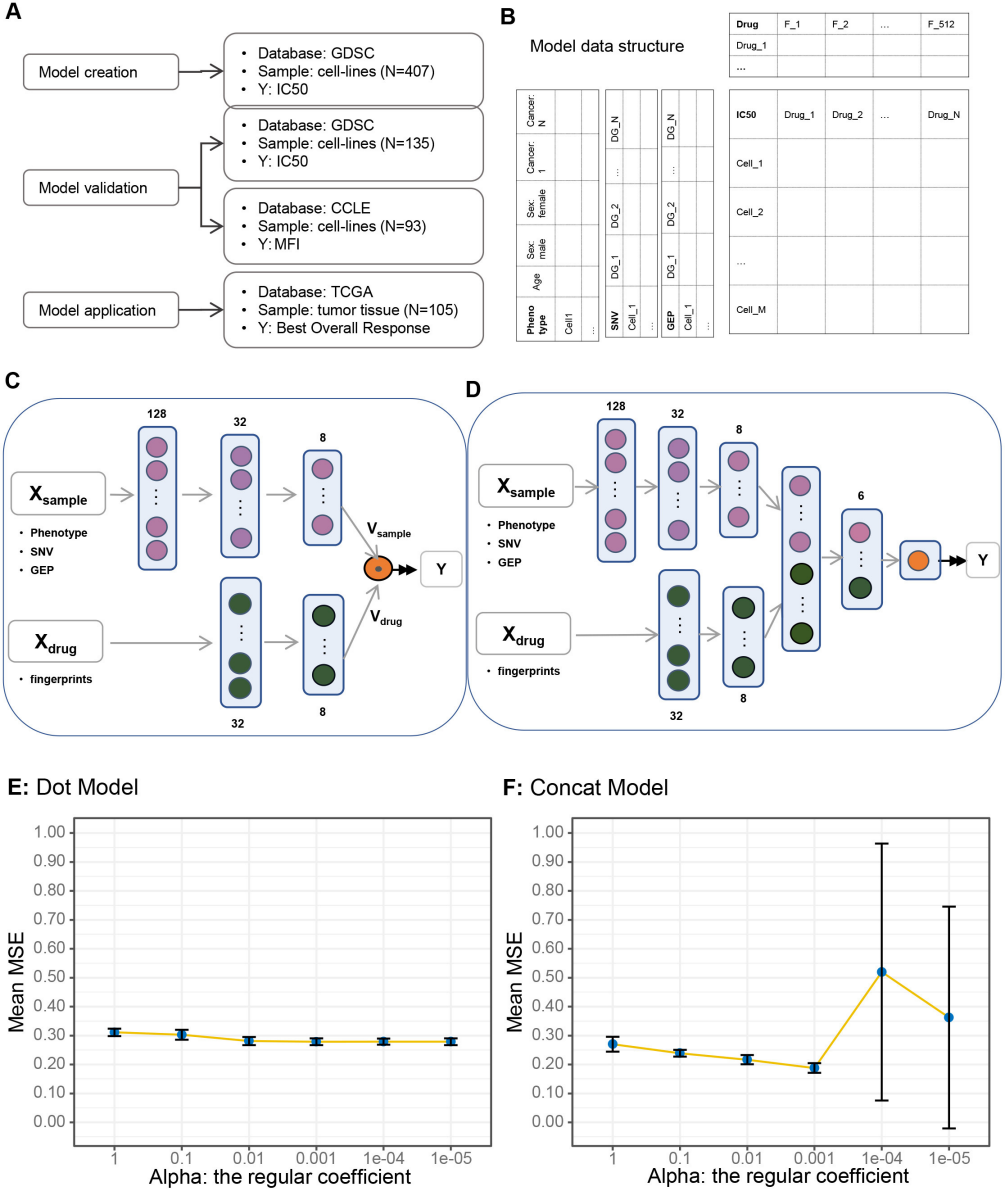
Study design and model description. **(A)** Study design and database usage. **(B)** Data structure. **(C)** Dot product (vector dot multiplication) algorithm. **(D)** Concatenate algorithm. **(E)** Model performance under dot algorithm: variations in model performance (mean square error) with changing regularization coefficients under the dot algorithm. **(F)** Model performance under concatenate algorithm.

We adopted a content-based filtering algorithm to construct the recommendation model. In our model, users represent cell lines, items represent drugs, and features include clinical characteristics, somatic mutation status (SNV), and GEP of cell lines and fingerprint of drugs. We constructed the model with multiple inputs consisting of the above features, with multiple dense layers, and with a single numeric output that represented the recommendation level (Figure 1; Supplementary Figure S1). The ReLU and linear activation function were used for hidden and output layers, respectively. Mean Squared Error (MSE) was used as the performance evaluation metric for our model, and the adaptive moment estimation (Adam) algorithm was used for optimization. The same L2 normalization was used for all layers, and the regularization coefficient was trained as a hyperparameter by a 10-fold cross-validation approach.

Model construction was implemented in jupyter notebook (version 6.5.2) on python (version 3.10.6) platform. Analysis software used in this study include: tensorflow (version 2.9.1), sklearn (version 1.1.3), rdkit (version 2022.09.1), pandas (version 1.5.1), numpy (version 1.23.4), pickle5 (version 0.0.11). Data visualization were conducted in *R* (version 4.4.0) with packages including ggplot2 (version 3.5.1), ggpubr (version 0.6.0), pheatmap (version 1.0.12), survival (version 3.6.4), survminer (version 0.4.9), and survivalROC (version 1.0.3.1)

## 3 Results

### 3.1 Construction of drug recommendation models

To build a drug recommendation model, we need data source, data structure, and model framework. We collected 542 GDSC cell lines with IC_50_ scores for 18 drugs from the GDSC database. To construct and validate the model, we performed the following steps to partition the data into training and validation sets: stratification based on cancer type and drug usage to ensure a balanced distribution of these two crucial factors, and random allocation of the data into a training set (*N* = 407) and a validation set (*N* = 135) in a 75:25 ratio. We then used cell lines (*N* = 93) in CCLE database as an independent validation dataset. The CCEL database uses MFI as drug sensitivity indicator, which differs from GDSC's IC_50_. Finally, to evaluate the performance of our model in real clinical scenarios, we applied the model to human tissue samples from the TCGA database (*N* = 105) by comparing the association between model-generated predictions and real BOR (Figure 1A).

The critical endpoint of the model is IC_50_, which is a matrix with each row representing a cell line, and each column representing a drug (Denck et al., 2023). The features of cell line and drug are documented as the left and top of the core matrix. Cell line features consisted of three integrated parts: the phenotype that include age, sex, and cancer type, the somatic mutation status (SNV) of known cancer driver genes (Supplementary Table S2), and the GEP of the same driver genes. Drug fingerprints with shape (512.0) were used as drug features (Supplementary Table S3; Figure 1B).

We constructed two model architectures using the same input data structure, namely the dot model and the concatenate model (Figures 1C, D; Supplementary Figure S2). The only difference between the two models was how to deal with the two vectors V_sample_ and V_drug_. In the dot model, we made a dot product of the two vectors, while in concatenate model we simply concatenated them together to a new vector and processed by one more dense layer.

To determine which architecture is better in drug recommendation, we trained the two models under the same hyperparameter alpha, the regularization coefficient, and compared the model metrics, i.e., the MSE using the 10-fold cross-validation setting (Figures 1E, F). The mean MSEs (Mean Square Errors) of the 10-folds decreased slightly in the dot model, while they decreased more quickly and then increased in the concatenate model, which implies that the concatenate model was more sensitive to alpha and was more accurate under optimal setting, i.e., alpha equals 0.001. Therefore, we chose the concatenated model with an alpha value of 0.001 as the foundational framework for our model.

### 3.2 Overview of model features in the GDSC dataset

**Figure 2:**
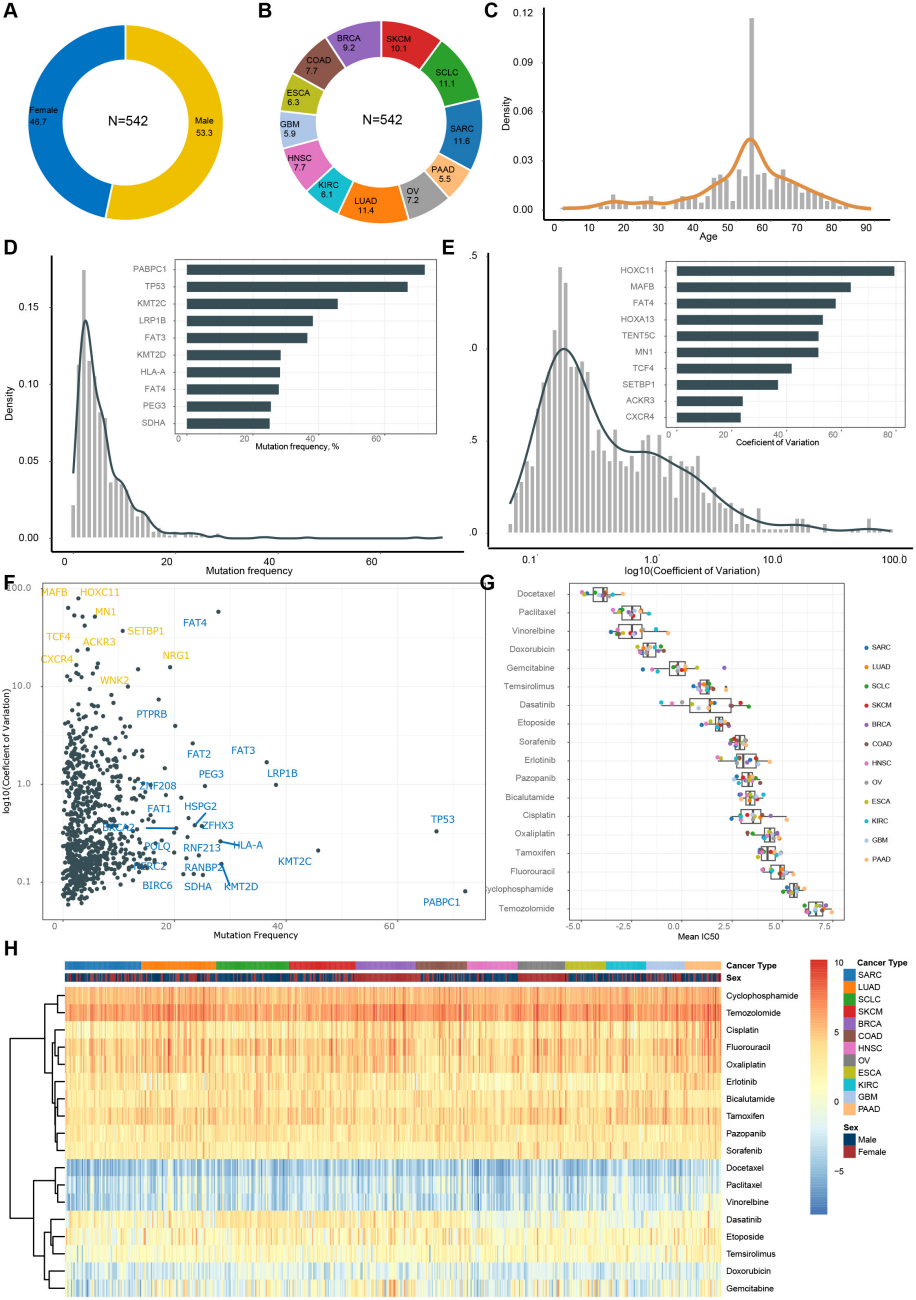
Features and endpoint description in GDSC dataset. **(A)** Gender composition. **(B)** Tumor type composition. **(C)** Age distribution. **(D)** Frequency of gene mutations (frequency of occurrence in samples). **(E)** Distribution of coefficient of variance for gene expression. **(F)** Mutations and expression of same genes. **(G)** Distribution of IC_50_ across different drugs. **(H)** Heatmap of IC_50_ within each patient.

In the GDSC dataset, we considered a comprehensive set of features for model construction, and these features played a pivotal role in the development of our predictive model. Our analysis encompassed 542 cell lines, including clinical attributes such as gender, age, and tumor type. Notably, there was a slightly higher proportion of males in the dataset, and the median age was 60 years (Figures 2A, C). Additionally, there were a total of 12 different tumor types (Figure 2B), with LUAD, SARC, SCLC, and SKCM having the highest sample counts, each exceeding 10%.

Regarding features related to the TME, we observed that PABPC1, TP53, and KMT2C exhibited the highest mutation frequencies (Figure 2D), while HOXC11, MAFB, and FAT4 displayed the greatest variation in gene expression (Figure 2E). These particular features likely made the most significant contributions to our model. It's worth noting that single nucleotide variations (SNV) and GEP complemented each other in deciphering the TME. Some genes exhibited high mutation rates, while others showed substantial expression variation. For instance, PABPC1 [Poly(A)-binding protein, cytoplasmic 1] is a gene that encodes a multifunctional protein involved in various cellular processes, including transcription and translation. It displayed a high mutation rate but low variance across samples, suggesting that its mutation status remained relatively consistent across different samples, despite mutations being present in the majority of samples. On the other hand, HOXC11 (Homeobox C11), a gene encoding a protein belonging to the HOX family of transcription factors critical for embryonic development and tissue positioning, had a low mutation rate but a high coefficient of variation. This indicated significant variation in mutation status among different samples, with some samples showing high variability.

Finally, we evaluated the sensitivity of various tumors to different drugs primarily using IC_50_ as the main indicator. Firstly, the most significant variation in drug sensitivity lies among different drugs (Figure 2G), with Docetaxel, Paclitaxel, and Vinorelbine showing the highest sensitivity, while Temozolomide, Fluorouracil, and others appear relatively less sensitive. Secondly, there is no apparent pattern of drug sensitivity differences among various cancer types for the same drug (Figure 2G). In other words, we did not observe any specific cancer type exhibiting markedly higher or lower sensitivity. Thirdly, substantial variations in drug sensitivity exist among different patients for the same drug (Figure 2H), particularly noticeable with Dasatinib, Temsirolimus, and Gemcitabine.

### 3.3 Training and evaluating model in GDSC dataset

**Figure 3:**
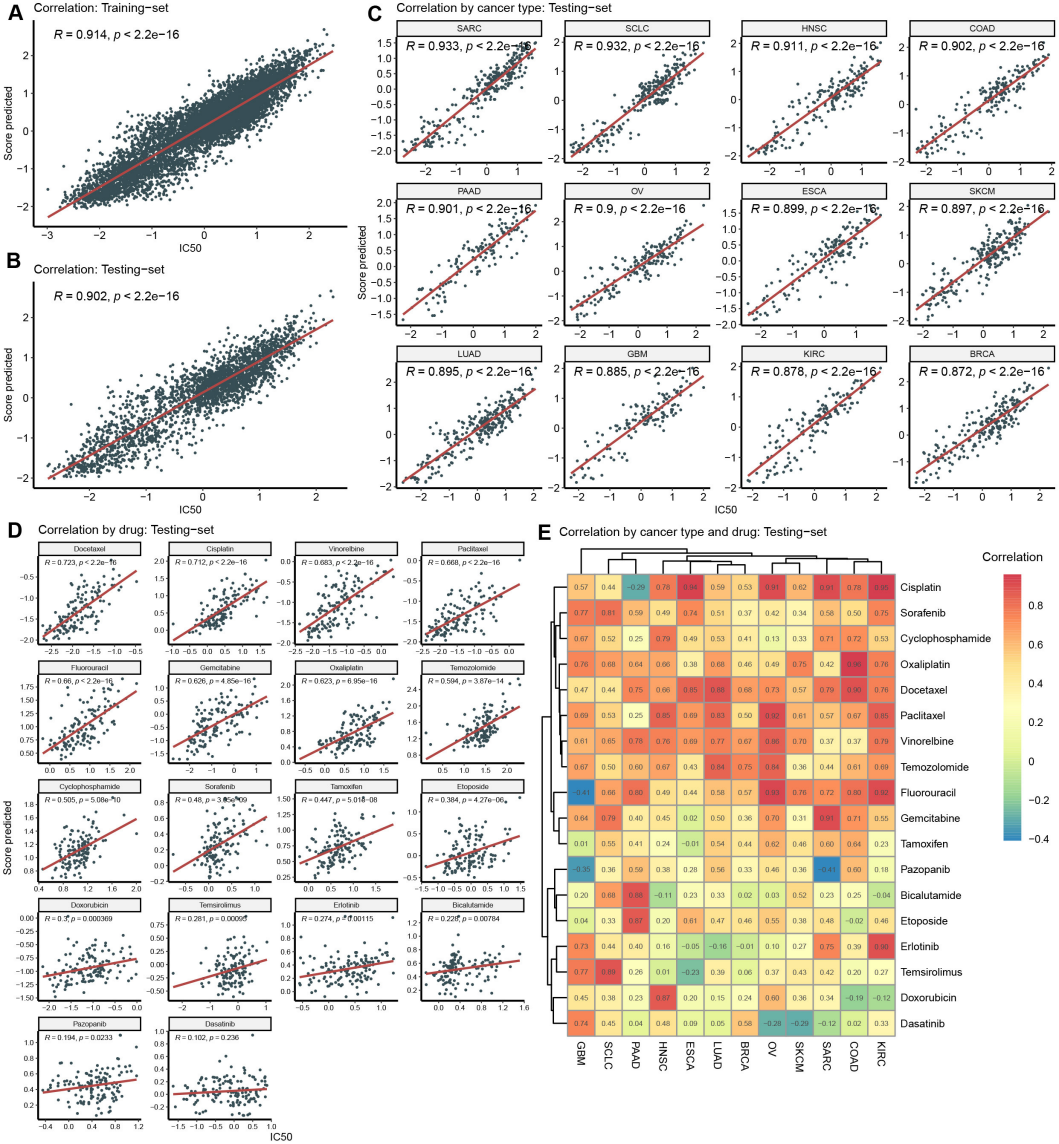
Model training and validation in GDSC. **(A)** Correlation between predicted score and actual IC_50_ in the training-set. **(B)** Correlation between predicted score and actual IC_50_ in the testing-set. **(C)** Correlation by cancer type in the testing-set. **(D)** Correlation by drug in the testing-set. **(E)** Correlation by cancer type and drug in the testing-set.

Utilizing the above features, we trained and validated the recommendation model in the GDSC dataset, with IC_50_ as response variable. The overall correlation coefficient (*R*) between the model-predicted scores and the actual IC_50_ in the training set and testing set was 0.914 and 0.902, respectively (Figures 3A, B). Regarding cancer type-specific evaluation, the correlation across all 12 cancer types in the testing set exceeded 0.85 (Figure 3C). Particularly in SARC, SCLC, HNSC, COAD, PAAD, and OV, the correlation surpassed 0.9, indicating a strong relationship between the predicted scores and actual IC_50_ Furthermore, we assessed the predictive performance of the model for different drugs within the testing set. In the correlation analysis of 18 drugs, varying degrees of correlation were observed (Figure 3D). Among them, Docetaxel, cisplatin, gemcitabine, and seven other drugs showed a strong correlation (*R* > 0.6), while Oxaliplatin, Tamoxifen, and 10 other drugs exhibited significant correlations with e IC_50_ (*P* < 0.05).

Deeply into predication accuracy in cancer type and drug specific way, we analyzed correlation analysis between predicted scores and IC_50_ in each pair of cancer-type and drug (Figure 3E). The numerical values within these heatmaps represent correlation coefficients. It's worth noting that the majority of these correlations are positive and exhibit relatively high values. For instance, in KIRC, SARC, OV, and ESCA, Cisplatin yielded positive correlations. However, in a few instances, negative correlations were observed. For negative correlations, one potential explanation could be the limited size of the training dataset, which may have constrained the model's ability to generalize effectively to these specific contexts.

### 3.4 Overview and comparison of model features in the CCLE dataset

**Figure 4:**
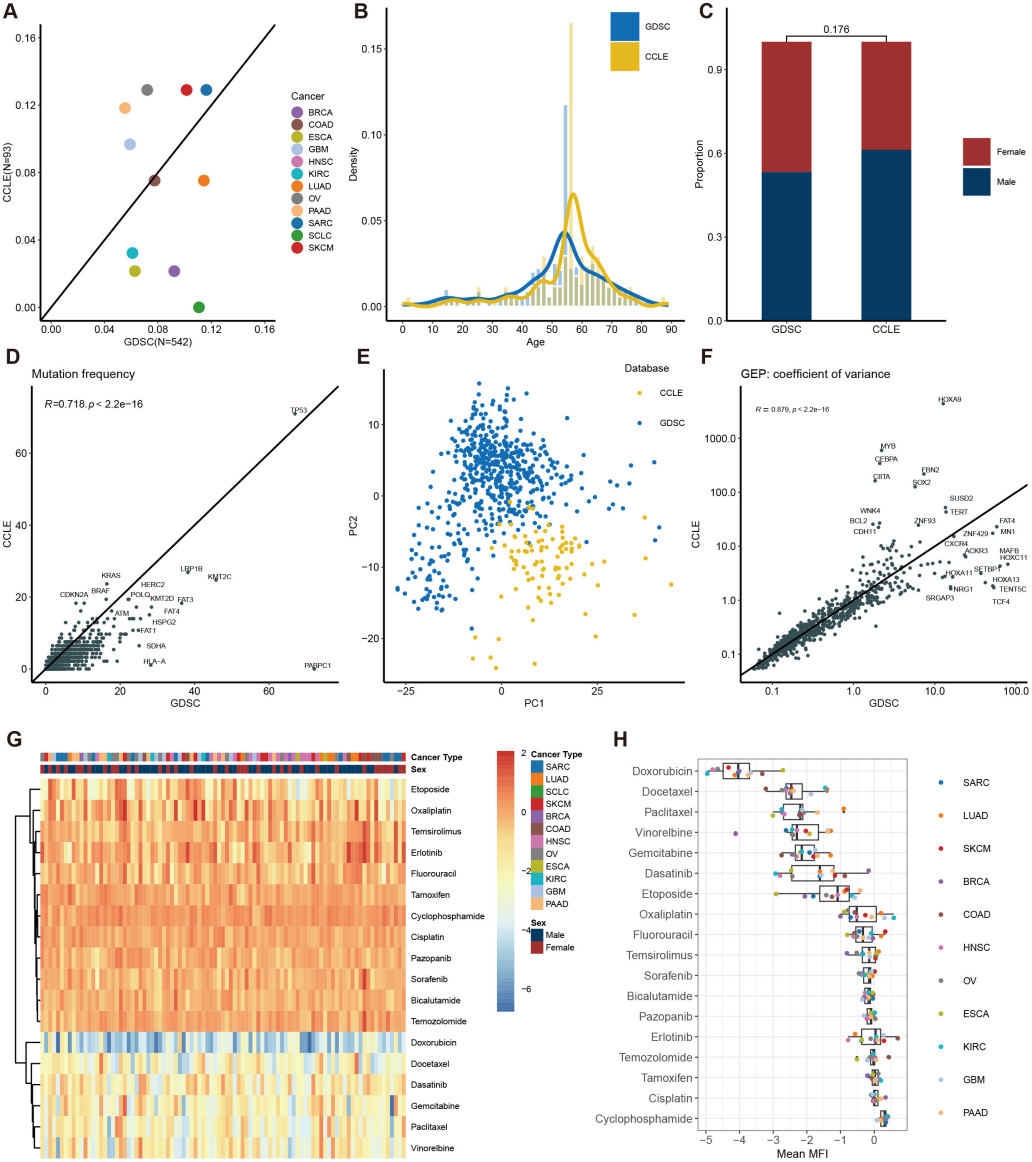
Description of features in the CCLE dataset and comparison with the GDSC dataset. Features concordance between CCLE and GDSC of cancer type **(A)**, age composition **(B)**, gender composition **(C)**, gene mutation frequency **(D)**, PCA of gene expression **(E)**, and coefficient variation of gene expression **(F)**. **(G)** Heatmap of MFI in each patient. **(H)** Distribution of MFI across different drugs.

To evaluate the generalization capacity of the model, we used the CCLE dataset for independent validation. First, we performed a comparative analysis of features between the CCLE and GDSC datasets. The analysis covered 12 different cancer types, revealing notable differences in the distribution priorities across the two datasets. SARC, SKCM, and COAD had similar proportions in both datasets, whereas BRCA, ESCA, and SCLC were more prevalent in GDSC but less so in CCLE. Conversely, OV, GBM, and PAAD were more heavily represented in CCLE but less frequent in GDSC (Figure 4A). These differences in cancer type distribution could potentially lead to suboptimal model training for cancer types underrepresented in GDSC. Figures 4B, C illustrate the composition of age groups and sex, respectively. Both datasets predominantly include individuals aged 45–70, and there's no significant difference of sex distribution was observed between the two databases. An intriguing observation is the consistent lower gene mutation rates in the CCLE dataset compared to GDSC, indicating that the cell lines in the CCLE dataset carry a lower mutational burden (Figure 4D). PCA analysis of gene expression data from CCLE and GDSC datasets reveals distinct clusters for each dataset, indicating their independence as separate data sources (Figure 4E). Nevertheless, the CCLE and GDSC datasets exhibit a high degree of correlation in terms of gene expression variability (Figure 4F).

Unlike the evaluation in GDSC, we utilized MFI as the primary endpoint to assess drug sensitivity in the CCLE dataset. While both measures assess drug sensitivity, it's noteworthy that in GDSC, Docetaxel had the lowest IC_50_ values (Figures 2G, H), whereas in CCLE, Doxorubicin displayed the lowest MFI values (Figures 4G, H). Of note, drugs like Capecitabine and Paclitaxel exhibited variations in drug sensitivity among different patients, highlighting the pronounced individual differences within the CCLE cell lines.

### 3.5 Evaluating model in the independent CCLE dataset

**Figure 5:**
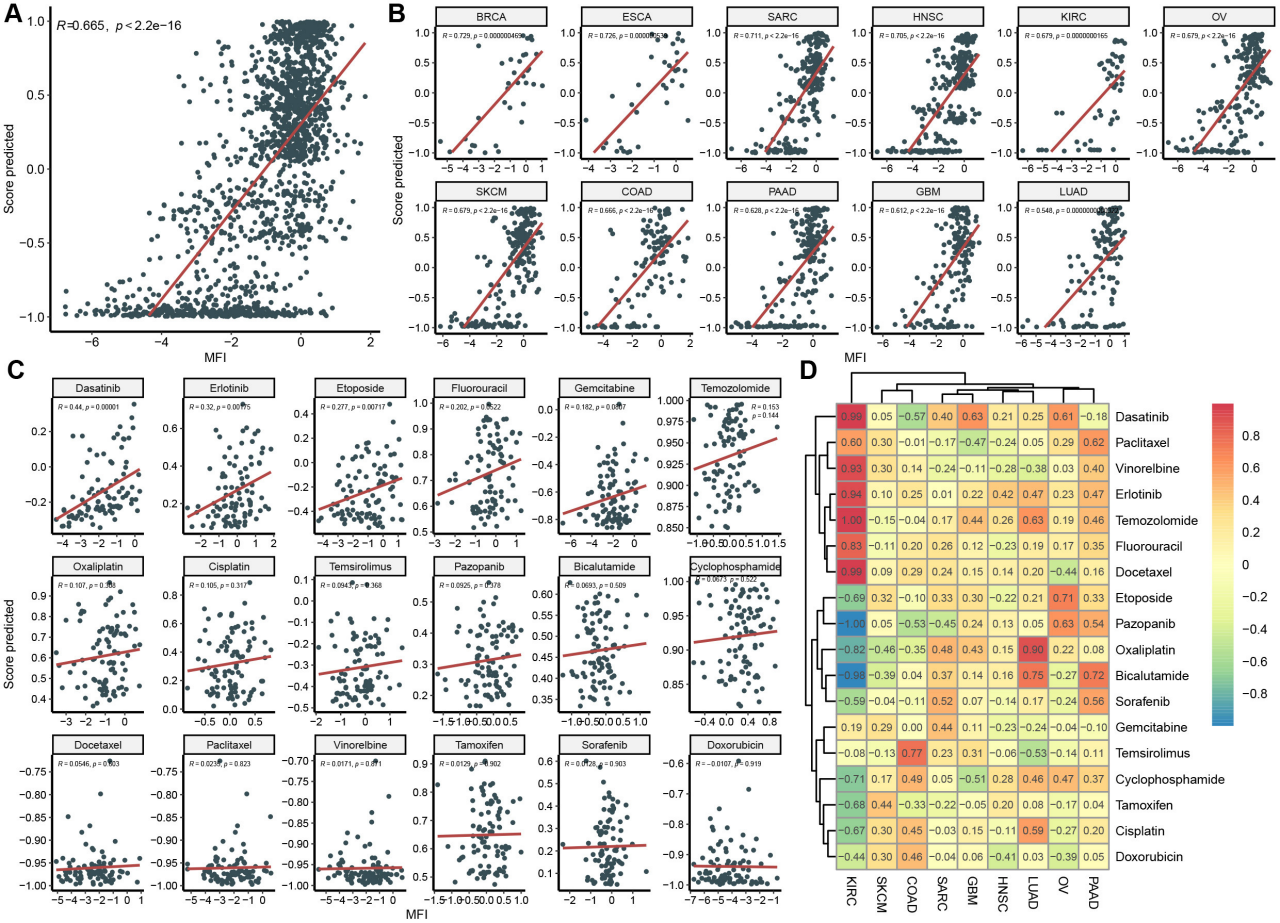
Independent model validation in CCLE Dataset. Correlation between predicted score and actual MFI in overall dataset **(A)**, in each cancer type **(B)**, in each drug **(C)**, and in each cancer type and drug pair **(D)**.

We independently validated our model using the CCLE dataset by assessing the correlation between the predicted scores and mean fluorescence intensity (MFI). Initially, we observed a strong correlation across the overall dataset (*R* = 0.67, Figure 5A). Given that some predictions deviated from actual MFI values, we conducted a more detailed analysis of correlations by cancer type and drug. Overall, the model performed well in a cancer-type-specific manner, with 11 out of 12 cancer types showing strong correlations (*R* > 0.6, Figure 5B). Specifically, the model performed best in cancer types with higher representation in the GDSC dataset, such as BRCA, ESCA, and SARC, but was slightly less accurate in underrepresented cancers like GBM and PAAD, which had a larger presence in the CCLE dataset. The model's performance in predicting sensitivity to individual drugs was suboptimal (Figure 5C), likely due to differences between IC_50_ and MFI measurements.

Like that in GDSC, we evaluated mode performance in a cancer type and drug specific way (Figure 5D). Correlation coefficient between predicted score and MFI in most cancer-type and drug pairs were positive, indicating an acceptable generalization performance of the model on an independent dataset with a different response variable. For instance, in KIRC, drugs including Dasatinib, Vinorelbine, Erlotinib, Temozolomide, Fluorouracil, and Docetaxel displayed strong positive correlations. However, there were some negative values, such as Pazopanib and Bicalutamide in KIRC, which maybe resulted from limited data in the cancer-type and drug pair.

### 3.6 Application of the model on patient data from TCGA dataset

**Figure 6:**
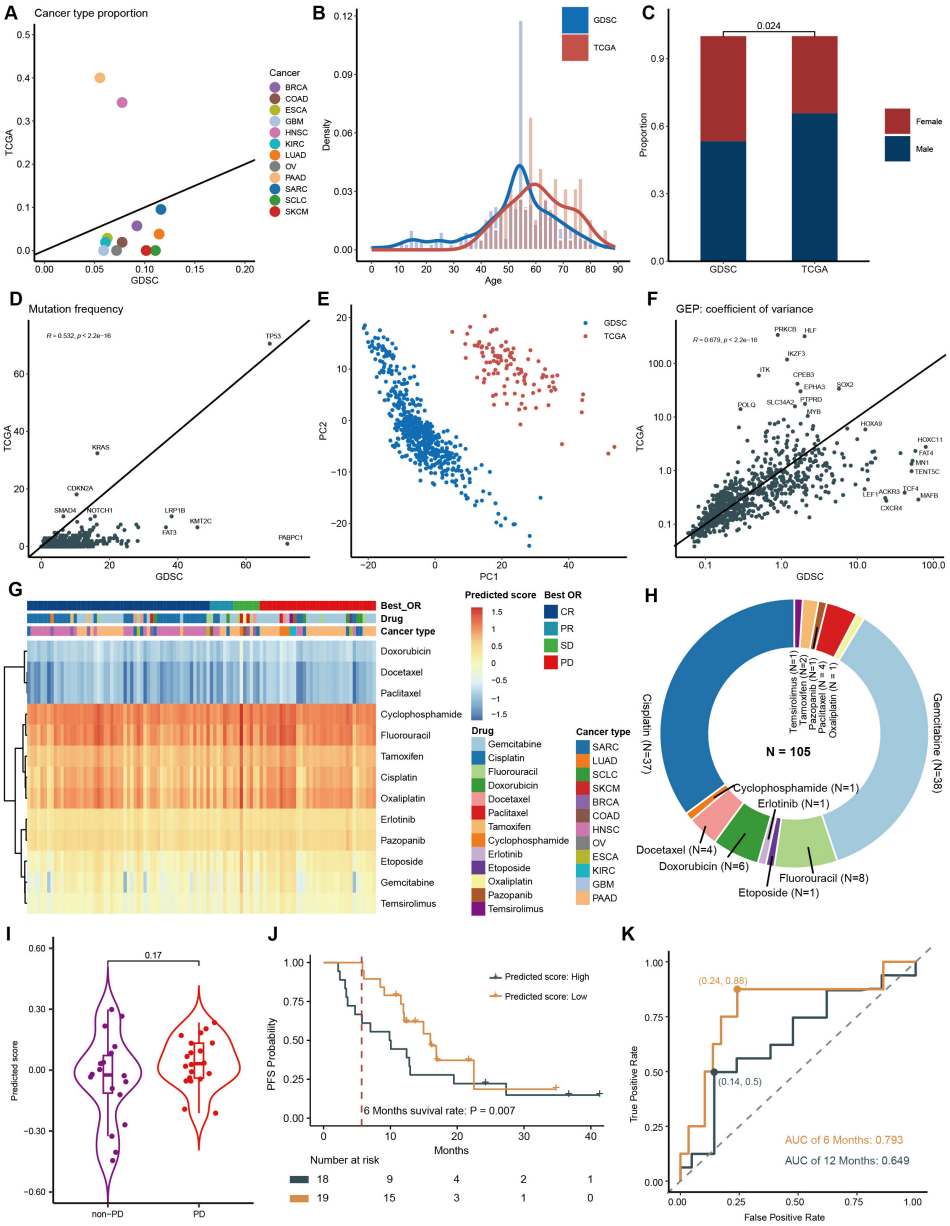
Model Application in TCGA patient data. Features concordance between TCGA and GDSC of cancer type **(A)**, age composition **(B)**, gender composition **(C)**, gene mutation frequency **(D)**, PCA of gene expression **(E)**, and coefficient variation of gene expression **(F)**. **(G)** Heatmap of predicted score. **(H)** Drug composition in TCGA dataset. **(I)** Comparison of predicting scores between PD and non-PD groups after gemcitabine treatment at pan-cancer level. **(J)** PFS curves and 6-month PFS rates for PAAD patients who received gemcitabine, categorized by high and low predicting scores. **(K)**. AUC curves for predicting 6-month PFS and 12-month PFS using model scores.

To assess the model performance in clinical scenario, we applied the model to TCGA dataset. We first retrieved 105 patient data from TCGA who received single-agent drug treatment, encompassing 12 tumor types and 18 drug categories, and then compared features between them and GDSC datasets. PAAD and HNSC are the two most prevalent cancer types in the TCGA dataset, but they are relatively underrepresented in the GDSC. In contrast, other cancer types have a lower prevalence in TCGA (Figure 6A). Notably, we observed a higher number of individuals aged between 60 and 80 in the TCGA dataset, while the GDSC cohort had more individuals aged between 10 and 40 (Figure 6B). Additionally, the proportion of males in the TCGA cohort was significantly higher than that in the GDSC cohort (Figure 6C).

Regarding gene mutation frequencies, the TCGA dataset displayed relatively lower mutation rates compared to GDSC (Figure 6D). We conducted Principal Component Analysis (PCA) on gene expression data from both TCGA and GDSC datasets. This analysis revealed that data points from the same database clustered closely together, while the differences between the two databases were substantial, confirming significant batch differences and the independence of these datasets as distinct data sources (Figure 6E). However, despite their different origins, we observed a high degree of correlation in gene expression variability between TCGA and GDSC datasets (*R* = 0.679) (Figure 6F), indicating consistent internal gene expression patterns across different datasets and further validating the broad applicability of our model.

By inputting the above parameters into the model, we obtained the predicted IC_50_ for each sample (Figure 6G). Consistent with findings from the GDSC dataset, drugs such as Docetaxel, Doxorubicin, and Paclitaxel exhibited the lowest predicted scores, indicating the highest sensitivity. Notably, drugs like Etoposide, Gemcitabine, and Temsirolimus displayed significant variations in sensitivity among patients, suggesting tumor heterogeneity. Since our cohort included patients who received only one drug, we were able to analyze the association between predicted IC_50_ and patients' BOR. Due to the predominance of patients receiving Gemcitabine and Cisplatin (Figure 6H) and the uneven distribution of BOR, only the Gemcitabine cohort was suitable for statistical analysis. At pan-cancer level, Gemcitabine tended to have a higher predicted score, indicating lower sensitivity, in the Progressive Disease (PD) group (*P* = 0.170, Figure 6I). Focusing specifically on PAAD, which is the cancer type that most frequently received monotherapy with Gemcitabine. When we divided patients into two groups based on the median of the predicted score, those with lower scores tended to have better progression-free survival (PFS) (HR = 0.572, 95% CI 0.26–12.6, *P* = 0.160, Figure 6J). Importantly, the 6-month PFS rate was significantly higher in the lower-score group compared to the higher-score group (0.9474 vs. 0.6111, *P* = 0.007, Figure 6J). Correspondingly, the predicting score accurately forecasted 6-month PFS (AUC 0.793; sensitivity 0.88; specificity 0.76, Figure 6K). However, its performance decreased significantly for 12-month PFS (AUC 0.649). This aligns with our understanding that drug sensitivity reflects immediate response rates, which can often translate into short-term survival, whereas long-term survival is influenced by various factors, including the patient's immune status.

## 4 Discussion

Drug recommendation models are designed to predict the response of cell lines or tumor tissue samples to various pharmacological agents based on their characteristics (Rafique et al., 2021). These models assist cancer researchers and clinicians in swiftly identifying the most suitable medication from a multitude of options, thereby enhancing the efficiency and personalization of cancer treatment (Qi and Zou, 2023). The models incorporate a wealth of clinical and omics features associated with the samples. Subtle individual differences can lead to diametrically opposite reactions to the same drug, necessitating the collection of diverse and representative data that reflect the variations among individual patients and the heterogeneity of tumors (Pinar et al., 2021; Khan et al., 2022; Xie et al., 2022).

For example, patient demographics such as age and gender can reveal disparities in cancer incidence across different sexes and age groups. The highest susceptibility to cancer is observed in individuals aged 50–70 (Figures 2A, C). As humans age, the accumulation of cellular mutations and metabolic changes may impact cellular growth and death, thereby increasing the risk of cancer. Omics characteristics can reveal changes at the genetic level, including the frequency of driver gene mutations and the variability of expression levels (Figures 2D–F). These genetic variations are crucial for understanding the molecular mechanisms of tumors, as they can help predict patient responses to chemotherapeutic drugs. By analyzing these gene mutations and expression patterns, researchers can target drugs to specific genes sensitive in different patients, better identifying those who may benefit from specific drug treatments.

To prevent model overfitting, L2 regularization was applied by introducing a penalty term to the loss function, which ensures the model avoids assigning excessively large weights to features. This approach promotes a simpler, more generalizable model that focuses on the most relevant characteristics, reducing noise from the training data. Additionally, L2 regularization improves stability by minimizing the model's sensitivity to small fluctuations in the data, leading to more consistent predictions. Using 5-fold cross-validation, we optimized the regularization strength (λ) within a range of [1, 1e-1, 1e-2, 1e-3, 1e-4, 1e-5]. Optimal performance was achieved at λ ≈ 1e-3, where the mean MSE was minimized (0.188) and standard deviation remained low (0.0167). Higher values of λ led to underfitting, while lower values introduced instability, underscoring the importance of appropriate regularization in optimizing model performance.

Performance validation is crucial for drug sensitivity prediction models. It serves multiple critical purposes: firstly, it ensures model credibility, enabling clinical practitioners and patients to trust the model's predictions (Mauvais-Jarvis et al., 2021). Secondly, performance validation aids in assessing a model's generalization capability, i.e., whether it can perform well on unseen data, which is pivotal for practical applications (Zhao et al., 2022; Krishnan et al., 2023). To achieve this, we split GDSC data into training and testing sets and investigated the correlation between predicted and actual values. The primary endpoint selected was IC_50_, with the performance metric being the Pearson correlation coefficient. High correlations (*R* > 0.9) were observed on both datasets (Figures 3A, B). Different cancer types may exhibit varying biological features, molecular mechanisms, drug targets, and drug sensitivities, all of which can affect the model's applicability, accuracy, and generalization capacity (Yu et al., 2021; Jin and Nam, 2021). Furthermore, diverse drugs may possess different modes of action, mechanisms, metabolic pathways, and side effects, influencing model complexity, interpretability, and translatability (Earl Hostallero et al., 2023; Chen and Zhang, 2022). In our correlation analysis based on cancer type and drug category, we made several noteworthy observations. When considering cancer type, we observed an excellent correlation among all 18 cancer types in the test dataset (Figure 3C). This indicated that our drug sensitivity prediction model maintained consistent and good performance across multiple cancer types. Secondly, when considering drug category, our analysis showed that 7 drugs had strong correlation (*R* > 0.6), and 17 drugs had significant correlation (*p* < 0.05) (Figure 3D). By further examining all possible combinations of 12 cancer types and 18 drugs (216 cases in total), we found that most cases showed positive correlation. This suggested that the predictive ability of our model could be extended to various cancer types and drugs. Notably, some drugs, such as Cisplatin, Paclitaxel, and Gemcitabine, exhibited correlation coefficients above 0.9 in specific cancer types, such as ESCA, SKCM, and SARC. These high correlations highlighted the potential of the model in identifying particularly effective therapeutic approaches for specific cancer types.

Utilizing different databases for validation helps evaluate a model's generalization capacity (Rong et al., 2021). To achieve this, we introduced CCLE as an independent database for model validation. We compared clinical and omics features between CCLE and GDSC (Figure 4). Significant differences were observed in gene mutation rates and principal component analysis, confirming the distinct nature of these datasets and ensuring the model's reliability and practicality across diverse backgrounds (Figures 4D, E). While the MFI endpoint was used in CCLE, IC_50_ was used in GDSC. IC_50_ measures the concentration at which a drug inhibits 50% of cell growth, providing a quantitative indicator of cytotoxicity. MFI, on the other hand, reflects a drug's inhibitory effect in a multiplexed high-throughput assay (He et al., 2022). Although the methodologies differ, both IC_50_ and MFI serve to quantify the cellular response to drugs, allowing us to draw meaningful comparisons regarding sensitivity. Different drugs may have different mechanisms of action on different cell lines, leading to varying sensitivities in GDSC and CCLE. For instance, Docetaxel is a microtubule stabilizer that inhibits microtubule dynamics, thereby preventing cell division and proliferation (Gambardella et al., 2022). Hence, it exhibits very low IC_50_ values in GDSC, indicating high sensitivity (Figures 2G, H). Conversely, Doxorubicin is a topoisomerase inhibitor that induces DNA damage and apoptosis. Consequently, it shows low MFI values in CCLE, indicating high sensitivity. However, in GDSC, Doxorubicin might not have a significant inhibitory effect on cell proliferation, resulting in higher IC_50_ values, indicating lower sensitivity (Figures 4G, H). These differences underscore the importance of considering the specific mechanisms of action of drugs and the context of the dataset when interpreting IC_50_ and MFI values in GDSC and CCLE. Despite the differences in features and endpoint measures between the two datasets, we still achieved strong correlations in CCLE, with an overall correlation coefficient of 0.67 (Figures 5B, C). In fact, this strong correlation held true in 10 out of 11 cancer types, and even in the remaining LUAD cancer type, while not exceptionally strong, a correlation coefficient of 0.55 was reached. However, when we analyzed the correlation between predicted values and MFI for individual drugs, we observed that most drugs did not exhibit significant correlations. Several factors might contribute to this observation. Firstly, it's possible that the differences between MFI and IC_50_ values play a role, as their association exists but does not follow a linear correlation pattern. Additionally, our sample size might be relatively small, leading to a wide distribution of cancer types for each drug. This aspect becomes apparent in the analysis of correlations among different cancer types and drugs. In most cancer types, the linear correlation for drugs hovers around 0.2. However, KIRC stands out as a unique case where some drugs, such as Dasatinib and Docetaxel, show a strong positive correlation, while others like Pazopanib and Bicalutamide exhibit a strong negative correlation. These findings emphasize the complexity of the relationship between predicted values and MFI in the context of different drugs and cancer types.

The use of cell lines for developing and validating drug prediction models offers significant convenience and cost-efficiency, facilitating in-depth exploration of molecular mechanisms and drug interactions (Wang et al., 2022; Feng et al., 2021). However, differences between cell lines and human patients, including genetic mutations, expression profiles, and epigenetic modifications, may affect the accuracy and reliability of these models in clinical applications (Singh et al., 2023). Therefore, integrating patient data from TCGA was essential to evaluate model performance in real-world clinical scenarios. Despite the observed differences in features between GDSC and TCGA datasets, the model demonstrated robust generalizability across various cancer types and age groups (Figures 6A–C). It is important to note that most patients in TCGA received combination therapies, making it difficult to isolate the effects of a single drug. To address this, we carefully selected patients who underwent monotherapy and used BOR as the clinical endpoint. While IC_50_ and MFI serve as laboratory-based drug sensitivity indicators, BOR directly reflects patient clinical outcomes, bridging the gap between preclinical and clinical data (Figures 6G–I). This cross-endpoint comparability demonstrates that, despite inherent differences between *in vitro* and *in vivo* measurements, our model can still provide consistent and accurate predictions. For patients treated with gemcitabine, Kaplan-Meier survival analysis revealed a significant difference in progression-free survival (PFS) between high and low predicted score groups. Patients with higher predicted scores showed notably lower 6-month PFS. The area under the curve (AUC) for 6-month survival was 0.793, indicating that the model's predictions not only capture drug sensitivity but also provide valuable insights into long-term survival, making it a promising tool for optimizing cancer treatment strategies.

It is important to note that while AI-driven models, such as ours, can offer valuable data-driven insights to aid clinicians, the responsibility for patient care must always reside with human healthcare professionals. AI models should function as decision-support tools, augmenting human judgment rather than replacing it. To ensure ethical and patient-centered decision-making, clinicians must have access to transparent explanations of the model's predictions, including any inherent limitations. Future efforts should focus on improving the interpretability of AI models in healthcare, enabling clinicians to fully comprehend the underlying mechanisms and rationale for the model's predictions, ensuring alignment with the best interests of the patient.

## 5 Conclusion

This study has successfully developed a drug recommendation model based on the TME and drug fingerprints, demonstrating high accuracy and stability in training and validation on the GDSC database. Furthermore, independent validation on the CCLE database and application to clinical patients in the TCGA database showcase the model's robust predictive performance across diverse data sources and drug sensitivity metrics, highlighting its potential value in real clinical scenarios. This work introduces a novel approach to precision cancer therapy, emphasizing the research's value and innovation. In summary, the paper provides new tools and methods for personalized cancer treatment, underscores the importance of model performance validation, and explores the heterogeneity between different data sources, offering substantial support for future cancer research and therapy.

## Data Availability

The original contributions presented in the study are included in the article/Supplementary material, further inquiries can be directed to the corresponding author.
